# Therapeutic Antibodies Against Shiga Toxins: Trends and Perspectives

**DOI:** 10.3389/fcimb.2022.825856

**Published:** 2022-02-10

**Authors:** Izabella de Macedo Henrique, Flavia Sacerdoti, Raissa Lozzardo Ferreira, Camila Henrique, Maria Marta Amaral, Roxane Maria Fontes Piazza, Daniela Luz

**Affiliations:** ^1^ Laboratório de Bacteriologia, Instituto Butantan, São Paulo, Brazil; ^2^ Laboratorio de Fisiopatogenia, Instituto de Fisiología y Biofísica Bernardo Houssay (IFIBIO Houssay-CONICET), Departamento de Fisiología, Facultad de Medicina, Universidad de Buenos Aires, Buenos Aires, Argentina

**Keywords:** Shiga toxin-producing *E. coli*, Stx toxins, antibodies, therapy, trends

## Abstract

Shiga toxins (Stx) are AB_5_-type toxins, composed of five B subunits which bind to Gb_3_ host cell receptors and an active A subunit, whose action on the ribosome leads to protein synthesis suppression. The two Stx types (Stx1 and Stx2) and their subtypes can be produced by Shiga toxin-producing *Escherichia coli* strains and some *Shigella* spp. These bacteria colonize the colon and induce diarrhea that may progress to hemorrhagic colitis and in the most severe cases, to hemolytic uremic syndrome, which could lead to death. Since the use of antibiotics in these infections is a topic of great controversy, the treatment remains supportive and there are no specific therapies to ameliorate the course. Therefore, there is an open window for Stx neutralization employing antibodies, which are versatile molecules. Indeed, polyclonal, monoclonal, and recombinant antibodies have been raised and tested *in vitro* and *in vivo* assays, showing differences in their neutralizing ability against deleterious effects of Stx. These molecules are in different phases of development for which we decide to present herein an updated report of these antibody molecules, their source, advantages, and disadvantages of the promising ones, as well as the challenges faced until reaching their applicability.

## 1 Introduction

Shiga toxins (Stxs) are potent cytotoxic proteins that can be produced and secreted by *Shigella dysenteriae 1* and by some serogroups of *Escherichia coli* (called Stx1 in *E. coli*), which also can produce a second type of Stx, called Stx2, antigenically distinct of Stx/Stx1, but with the same action mode (Melton-Celsa and O'Brien, 2014). Shiga toxin-producing *Escherichia coli* (STEC) is a family of bacteria that share the possibility to secrete Stx. STEC are foodborne pathogens that may colonize and damage the human colon, where they secrete Stx that gain access to the bloodstream and damage different target organs: mainly kidney and brain. Indeed, STEC infection may develop hemolytic uremic syndrome (HUS) because of Stx in the target organs.

Currently, there are no protective measures or therapies against Stx intoxication, and the treatment is solely supportive and includes rehydration therapy, and, where necessary, dialysis. The neutralization of Stx before the appearance of HUS severe symptoms is one of the promising approach; therefore, this review summarizes one of the most studied neutralization molecules, the antibodies.

Antibodies are key molecules for therapeutic proposal. Its modular structure enables it to be engineered to have tags for purification and immunoprecipitation, conjugation sites to improve chemical space, or mutagenesis to map the CDRs, which allow affinity improvement (Gitlin, 1966; Tiller and Tessier, 2015; Basu et al., 2019). As recombinant molecules, they can be produced by several distinct hosts, such as mammalian, insect, yeast, and bacterial systems, the last one requires low-cost production media and equipment, besides being able to synthesize and express practically unlimited amounts of antibodies to almost any antigen, therefore, an interesting molecule used either by industry or academia, ranging from bench to large-scale production (Robinson et al., 2015).

The main aim of this review is to provide an update regarding antibodies raised towards Stx to prevent their toxic effects, by contextualizing the intoxication problem and how antibodies can be used as a therapeutic approach to solve it.

### 1.1 Shiga Toxins and Their Toxic Outcomes

Stxs are the main virulence factor of STEC and are responsible for developing HUS. Stx is encoded in the late region of the genome of lambdoid prophages integrated into the bacterial chromosome (O'Brien et al., 1983) and is optimally expressed after the induction of the lytic cycle. In this regard, Stx phages constitute an important lateral gene transfer mechanism that may contribute to the emergence of new STEC strains (Schmidt, 2001; Bielaszewska et al., 2014). An example of this gene transfer occurred in the outbreak in Europe in 2011 where an enteroaggregative *E. coli* strain acquired the Stx phage, and this newly STEC strain affected mainly adults (Borgatta et al., 2012).

Stx can be classified in antigenically different types and subtypes: Stx (from *Shigella* sp.), Stx1 (Stx1a, Stx1c, Stx1d, Stx1e), and Stx2 (Stx2a, Stx2c, Stx2d, Stx2d_act_, Stx2e, Stx2f, Stx2g, Stx2h, Stx2i, Stx2j, Stx2k—recently reported but have yet to be broadly accepted—and Stx2l). Stx subtypes differ in their amino acid sequence, and an analysis of the Stx protein sequences showed that Stx1, Stx1c, and Stx1d have 93%–100% homology and Stx2a to Stx2g have also high homology (93%–100%), except for Stx2 and Stx2f (69%) (Golshani et al., 2016). Among the several Stx subtypes, the prototype toxin for each group is now designated Stx1a or Stx2a (Melton-Celsa and O'Brien, 2014).

All Stx types and their subtypes are AB_5_ toxins characterized by the presence of a one active A domain (~32 kDa) that blocks cell protein synthesis by cleavage of ribosomal RNA, and a pentameric binding domain B (~7.7 kDa each), with close affinity to the glycosphingolipid globotriaosylceramide (Gb_3_, CD77) and, to a lesser extent, globotetraosylceramide (Gb_4_) (Legros et al., 2018), which are found in a variety of human cells, such as glomerular and brain endothelial cells. Receptor-mediated internalization of the toxin results in the inhibition of protein synthesis, ribotoxic stress that finally leads to apoptosis (Tesh et al., 1993; Melton-Celsa and O'Brien, 2014).

Not all Stx subtypes have been associated with severe illness (Beutin et al., 2004; Hofer et al., 2012). In this regard, it has been described that each Stx variant differs in pathogenicity. While Stx1a strains are associated with hospitalization and bloody diarrhea, Stx1c and Stx1d are less often associated and not enough information is available about the clinical significance of Stx1d (Kumar et al., 2012; EFSA BIOHAZ Panel et al., 2020). On the other hand, Stx2a and Stx2c are clinically more related with severe cases of HUS, and additionally, it is generally described that Stx2a expressing STEC strains develop more severe cases of HUS with a higher risk of encephalopathy (Orth et al., 2007). Differently, Stx2d and Stx2e are associated with milder or asymptomatic infections (Friedrich et al., 2002; Orth et al., 2007). Stx2e strains are not often found in STEC infections associated with human disease, and Stx2f strains have recently been isolated from patients with HUS (Friesema et al., 2015; De Rauw et al., 2018). Stx2g subtype is also rarely associated with human illness and not usually associated with severe illness (Prager et al., 2011).

**Figure 1:**
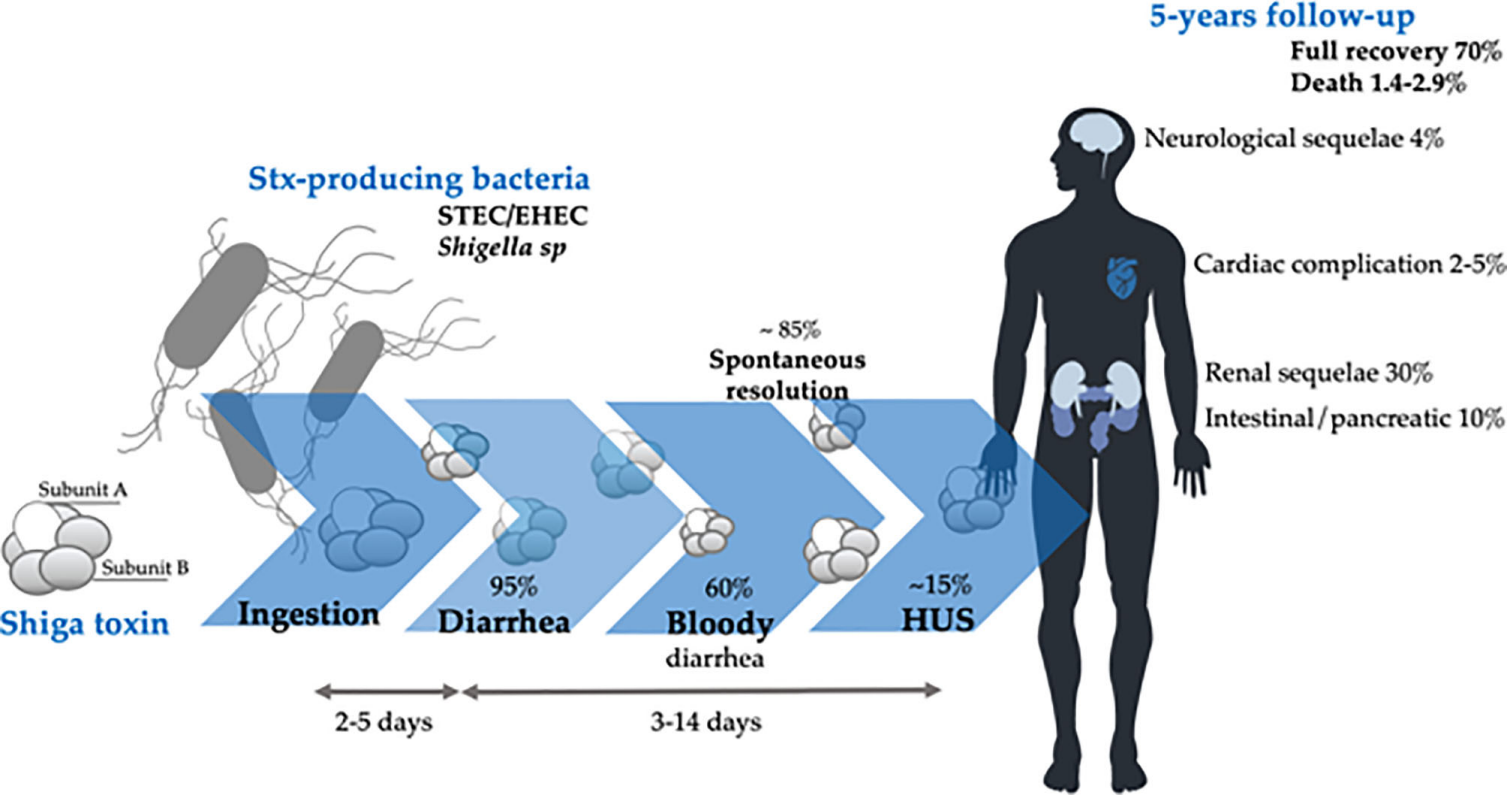
Clinical course and main outcomes of Stx-producing bacterial infection. Modified from (Bruyand et al., 2018).

The pathology associated with the Stx toxicity starts with Stx-producing bacterial infection. **Figure 1** summarizes the disease course and main outcomes of Stx intoxication, which begins with diarrhea that can be self-limited. In some cases, it may evolve to bloody diarrhea, hemorrhagic colitis (HC), and, in approximately 15% of the infections (Tarr et al., 2005), develop HUS characterized by thrombocytopenia, microangiopathic hemolytic anemia, and acute kidney injury (Repetto et al., 2013).

Development of HUS occurs approximately 7 days after the onset of gastrointestinal symptoms and 4 days after the onset of bloody diarrhea (Bitzan, 2009). Intervention strategies for blocking Stx, as primary therapy for directly preventing HUS development, may be applied in this period between STEC infection and before the appearance of HUS symptoms.

The clinical features of cytotoxic effects of Stx are determined by the damage to endothelial cells of small vessels mainly localized in the colon, kidney, and central nervous system (CNS) (Richardson et al., 1988); however, several other organs can be affected, such as pancreas and liver, and consequently, endothelial damage can be widespread in the microvasculature (Luna et al., 2021). Nevertheless, not all cells undergo cell death upon binding and uptake of Stx. Toxin binding to platelets, leukocytes, and erythrocytes can lead to their activation without inducing cytotoxicity (Karpman and Ståhl, 2014). During activation, cells release microvesicles (MVs) and it has been described that Stx may be released within MVs (MVs-Stx) from blood cells during HUS (Ge et al., 2012; Arvidsson et al., 2015; Ståhl et al., 2015). So, these MVs-Stx can transport Stx into the kidney, evading the immune system, and contributing to kidney failure in HUS patients (Ståhl et al., 2015).

The kidney is seriously affected by Stx because of the presence of specially Stx-sensitive cells that express high amounts of Gb_3_ receptor as microvascular endothelial cells that express 50-fold higher Gb_3_ levels than macrovascular endothelial cells (Obrig et al., 1993; Obrig, 2010). Moreover, because of the high volume of blood flow and filtration rate, the possibility of Stx interaction with cells of renal microvasculature and the filtration barrier increase (Obrig, 2010). The thrombotic microangiopathy lesion is the typical injury caused by Stx in the kidney because of the direct action of Stx on glomerular endothelial cells that consists of thickening of arterioles and capillaries, swelling and detachment of endothelial cells from the basement membrane, and platelet thrombi that obstruct the microcirculation (Zoja et al., 2010). Inflammation also can contribute to endothelial damage. In this sense, Stx induces the release of pro-inflammatory cytokines, leukocyte recruitment, platelet aggregation, and fibrin deposition. All these events lead to partial or complete vessel occlusion by microthrombi and the consequent microangiopathic hemolytic anemia (Exeni et al., 2018). The kidney injury is expressed as different degrees of renal failure (Repetto, 2005; Repetto et al., 2013), and the endothelial dysfunction is essential to the development of microvascular lesions in HUS (Morigi et al., 1995; Ruggenenti et al., 2001).

The main effects of Stx on the endothelial cells are intracellular edema and a decrease of cell viability by apoptosis and necrosis (Amaral et al., 2013). Likewise, Stx also causes damages to renal epithelial cells by the inhibition of protein synthesis, apoptosis, and necrosis, showing the direct effect of this toxin on the renal tubules (Karpman et al., 1998; Kaneko et al., 2001; Creydt et al., 2006).

Moreover, there is evidence that a direct effect of Stx is also on the central neural system (Obata et al., 2008). It was demonstrated that Stx2 has a direct action in the brain of rats since it produces damage in neurons, astrocytes, oligodendrocytes, and endothelial cells (Goldstein et al., 2007). Furthermore, Stx2 breaks the blood-brain barrier (BBB) and damages cells that modulate motor functions (Pinto et al., 2017). Stx2 may act through Gb_3_ neuronal receptors, and this toxin was detected inside neurons that upregulated the Gb3 receptor (Tironi-Farinati et al., 2010).

### 1.2 Stx-Producing Bacteria

STEC and its subgroup enterohemorrhagic *E. coli* (EHEC) are the major causes of gastroenteritis and competent Shiga-toxin producers. Worldwide, around 2.8 million people were infected per year, and over 250,000 illnesses occurred in the USA due to STEC, and although several serotypes are harmful, one-third originate from the O157:H7 serotype (Bird, 2019).

In Latin America, the southern countries are the most affected, especially Argentina. There, HUS is endemic, and although Argentina has the greater incidence rates in the entire world, the correlation of infection rates between countries is problematic since each country has unique testing parameters that make this evaluation difficult; a preoccupation due to the outbreaks in Argentina also relays the relevant role of the nation in the bovine meat exportation (Torres et al., 2018; Torti et al., 2021). For that reason, meaningful research in this field is done in Latin America (Torres et al., 2018).

Majority of STEC infection occurs through fecal-oral contamination, contaminated water, and food consumption, such as undercooked meat (below 71°C), unpasteurized food, contaminated vegetables, and person-to-person contact (Alconcher et al., 2020). Cattle are the principal reservoirs for STEC, and the bacteria can survive for months in soil, water, or organic material (Sapountzis et al., 2020).

Upon ingestion, STEC/EHEC resides in the intestinal tract and adheres to the gut epithelium of the distal ileum and colon. Fimbriae promote an initial binding, which, in EHEC infections, is followed by the effector protein (Esp proteins) injection by a type III secretion system (T3SS) (Donnenberg and Kaper, 1992; Garmendia et al., 2005; Gaytán et al., 2016). After injection of the translocated intimin receptor (Tir) into the host cell plasma membrane, it interacts with the bacterial outer membrane protein intimin, triggering the intimate attachment to the host cell and the effacement of the brush border microvilli, initiating actin polymerization and subsequent formation of attaching and effacing (A/E) lesions. The genes encoding Tir, intimin, and the T3SS are localized on the chromosomal locus of enterocyte effacement (LEE) pathogenicity island present only on EHEC strains. LEE-negative STEC strains may also produce severe disease since other adhesins may be involved in adhesion/colonization of this subset of bacteria to enterocytes (McWilliams and Torres, 2014) and from the unusual HUS-inducing *E. coli* strain EAEC of serotype O104:H4 bearing *stx2* gene, which was responsible for the major outbreak in Germany and parts of Europe in 2011 (Bielaszewska et al., 2011; Mellmann et al., 2011).

Shigellosis is bacillary dysentery caused by *Shigella*; these bacteria, at first, attack epithelial cells of the large intestine, and then the infection reaches nearby cells. Transmission can happen by the fecal-oral route, also due to contaminated food and water, or through vectors like flies. *Shigella* as STEC has the incredible characteristic of causing an illness with a low infection dose of only 100 organisms. This characteristic makes *Shigella* a danger to human health and a significant food safety concern since this pathogen has been responsible for so many epidemics (Lampel et al., 2018).

There are four serogroups; *S. dysenteriae* with 12 serotypes; *S. flexneri* with 6 serotypes; *S. boydii* with 18 serotypes; and *S. sonnei* with 1 serotype, also known as A, B, C, and D, respectively (Hale et al., 1996).

The serogroups A and B are the promoters of endemic and epidemic shigellosis (respectively) in developing countries, with high transmission rates and significant cases of fatality rates. Only A can cause infection; C and D serogroups are mild, causing watery or bloody diarrhea (Williams and Berkley, 2018). However, in general, *Shigella dysenteriae* type 1 strains are the most reported to be associated with Stx toxin production (Mark Taylor, 2008; Williams and Berkley, 2018).

## 2 Antibodies as Therapeutic Tools

Antibodies are ubiquitous molecules, and because of their ability to highly recognize and bind to an antigen, besides mediating the interaction with other cells and molecules of the immune system, they have been used in a wide range of biotechnological approaches (Basu et al., 2019).

**Figure 2:**
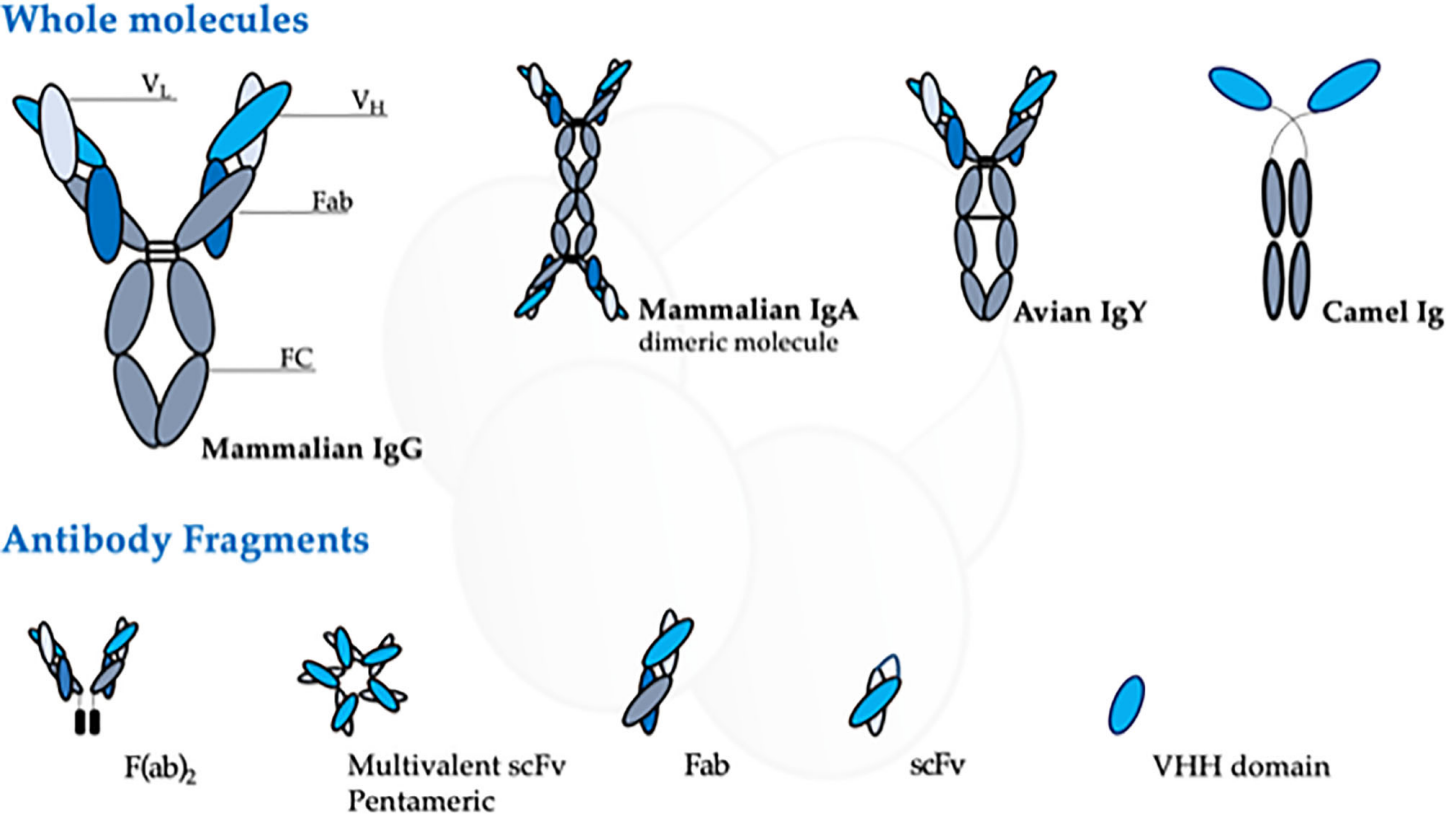
Schematic representation of the different antibody source, structure, and arrangement presented at this review. The figure highlights the domains of the mammalian IgG, the most used antibody molecule.

Although the mammalian immune system can produce five classes of immunoglobulin, the IgG is the most used for antibody research. It has a “Y”-shape structure consisting of two light and two heavy chains that form a conserved crystallizable fragment (FC), responsible for effector functions of the antibody, and an antigen-binding site (Fab), which determines the molecule specificity (Nilvebrant and Sidhu, 2018) (**Figure 2**). The Fab fragment contains three hypervariable regions called complementary domain regions (CDR) responsible for hypervariability and specificity against different antigens.

The versatility and specificity of these molecules attract significant attention for their use as therapeutic tools. Indeed, the global market for monoclonal antibodies is projecting a compound annual growth rate (CAGR) of 5.3% between the years 2020 and 2026 and its prospect to reach the value of 11.77 billion dollars in 2026 (Kaplon and Reichert, 2021). There are numerous motivations to be interested in antibodies. From the business view, engineered antibodies represent potential solutions to challenges facing the industry, including the shortage of innovative candidates in the pipeline and low approval success rates for new therapeutics.

The comparatively high approval success rates for monoclonal antibodies (mAbs), for example, are probably one reason for the growing interest in the development of these therapeutics. As described by Hay et al. (2014), biologics such as mAbs have a probability of approval of near 1 in 4 compared with that of new molecular entities, which is nearly 1 in 8. Basically, mAbs are granted marketing approvals at twice the rate of small molecule drugs (Kaplon and Reichert, 2019). On the other hand, from the medical science perspective, innovative protein engineering allows for the design of antibody molecules with decreased immunogenicity, enhanced effector functions, and improved pharmacokinetic properties (Reichert, 2009).

To generate antibodies for therapeutic application, there are three main approaches: (i) by animal immunization to generate polyclonal antibodies (pAbs); (ii) by lymphocyte immortalization creating single clones secreting specific mAbs; and (iii) by DNA recombinant technology and heterologous expression system creating diverse recombinant (rAbs) antibody formats for different goals (Cosson and Hartley, 2016).

Following, we provide a review of the antibodies raised against Stx and their different presentation (**Figure 2**) to update the efforts towards the search for an effective neutralizing molecule against the most severe Stx intoxication symptoms.

### 2.1 Antibodies Against Shiga Toxin Activity

**Figure 3:**
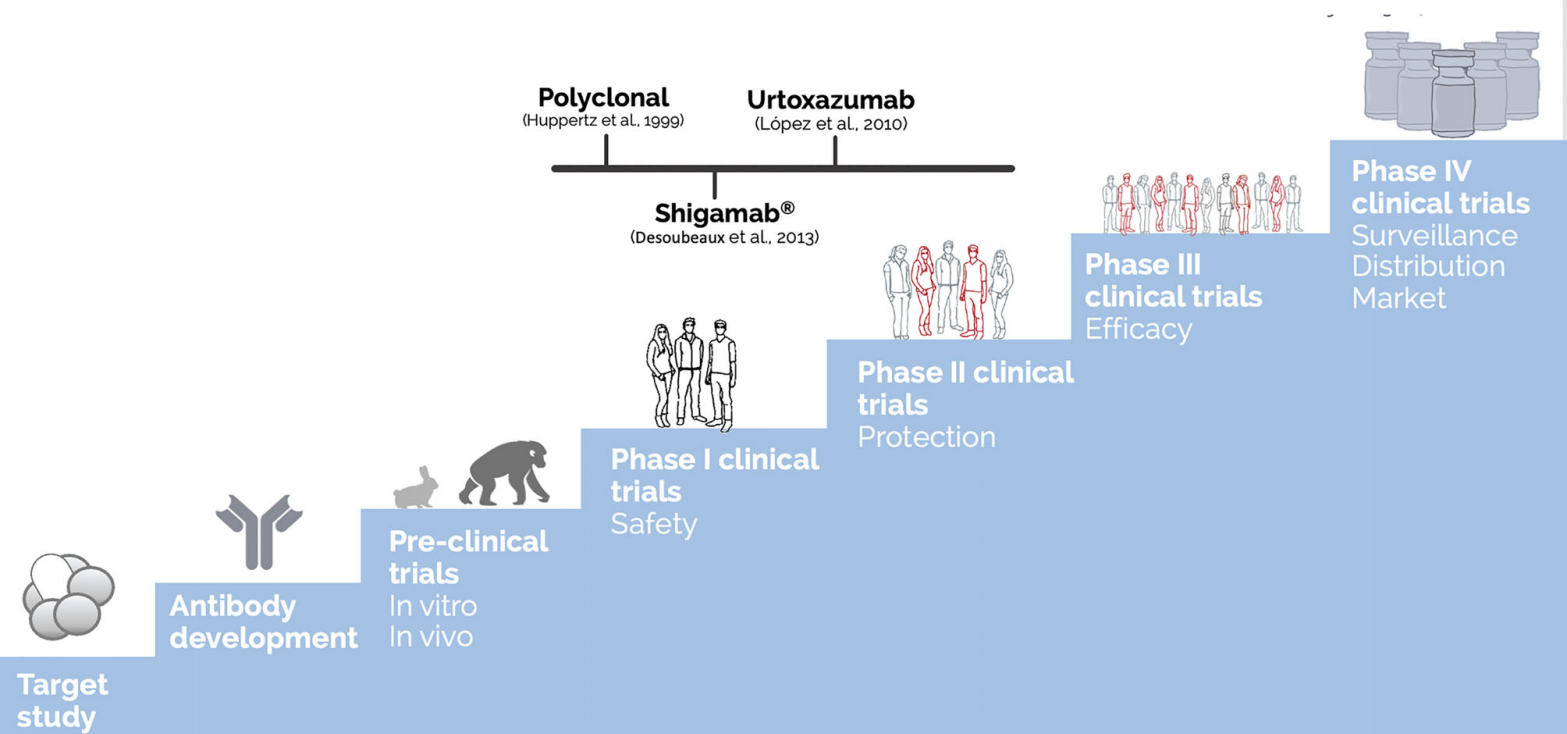
Development steps of biopharmaceuticals until human use approval and the most advanced antibodies raised against Stx. The discovery of a new drug as therapeutic antibodies starts with the target study followed by the antibody development (choosing of which antibody, type, format, avidity, and affinity) and achieving the functional test *in vitro* and *in vivo* (using different animal models, starting with mice and rabbits until the most closely related to humans as apes), reaching the challenge assay in an animal model, which is part of the preclinical trials. Once the molecule shows relevant ability to neutralize the effect of the target, human clinical trials could be performed. The clinical trials are generally divided into four phases: The first one enrolls dozens of volunteers, and the main endpoint is to test the safety of the molecule. By phase II, the molecule is tested in hundreds of volunteers to attest protection towards the toxin effect. At the last phase before regulatory agency approval, controlled randomized double-blind assays are performed in millions of volunteers to test the molecule efficacy. Once passed at those clinical trials, the therapeutic molecule can be approved by the regulatory agencies to be administered in humans, which is considered phase IV, in which the surveillance of its long-term administration is going to take place (Tamimi and Ellis, 2009). The polyclonal developed by Huppertz (Huppertz et al., 1999) and Shigamab^®^ (Desoubeaux et al., 2013) tested safety as a phase I clinical trial. On the other hand, urtoxazumab tested safety and pharmacokinetic, which could be considered a clinical trial 1/2 (López et al., 2010).

Before becoming a biopharmaceutical, any therapeutic molecule, from vaccine candidates to antibodies must get through several testing stages to ensure that the final compound is safe and efficient for human administration. These steps include establishing the pharmacology and biochemistry of the molecule of interest through various *in vitro* and *in vivo* tests to assess its safety (Tamimi and Ellis, 2009). **Figure 3** summarizes these steps and presents the most advanced antibodies raised against Stx, which reached clinical trials. Moreover, **Table 1** summarizes all the anti-Stx antibodies cited from basic polyclonal until innovative recombinant antibodies, their format, and stage of development.

**Table 1:** List of antibodies included on this paper and their development stage.

Reference	Name		Target	Molecular format	Source	Development stage
Mohawk et al. (2010); Kokai-Kun et al. (2000)	Anti-Stx2 antiserum		Stx2	Polyclonal	Rabbit serum	Preclinical
Takeda et al. (1993)			Stx1; Stx2	Polyclonal	HUS patient and commercial serum	*In vitro*
Fujii et al. (1998)			Stx2	Polyclonal	Rabbit serum	*In vivo*
Huppertz et al. (1999)			Stx1; Stx2	Polyclonal	Immunoglobulin-rich bovine colostrum	Clinical trials
Funatogawa et al. (2002); Seita et al. (2013)			Stx1; Stx2	Polyclonal	Colostrum IgG	*In vivo*
Garimano et al. (2021)			Stx2	Polyclonal	Hyperimmune bovine colostrum	*In vivo*
Parma et al. (2011) (Tironi-Farinati et al., 2010)			Stx2B; Stx2	Polyclonal IgY	Egg yolks	*In vivo*
Feng et al. (2013)			Stx2e	Polyclonal IgY	Egg yolks	Preclinical
Fathi et al. (2020)			Stx1; Stx2	Polyclonal IgY	Egg yolks	Preclinical
Padhye et al. (1989)		3C10^a^; 4c9^b^; 5E1^b^; 6Fl^b^; 9c9^a^; 10D11^a^; 10D12^b^; 10F4^b^; 1C5^a^; 1E1^b^; 2D5^b^; 3E4^b^; 4F10^b^; 8H10^b^	Stx1A/B; Stx2A/B	Monoclonal IgG/IgM	Hybridoma	*In vitro*
Nakao et al. (1999)	VTm1.1		Stx2 B	Monoclonal IgG	Hybridoma	*In vitro*
Nakao et al. (2002)	5-5B; 6-5C; 13-3E; 13-5C;18-6D		Stx1 B	Monoclonal IgG	Hybridoma	*In vitro*
Ma et al. (2008)	5F3 and 5C11, 1A4 and 1A5		Stx2 A; Stx2 B	Monoclonal IgG	Hybridoma	*In vivo*
Rocha et al. (2012)	3E2 and 2E11		Stx1; Stx2	Monoclonal IgG	Hybridoma	*In vitro*
He et al. (2013); Russo et al. (2014); Skinner et al. (2015)	Stx2-1; Stx2-2; Stx2-4; Stx2-5; Stx2-6		Stx2	Monoclonal IgG	Hybridoma	Preclinical
Ma et al. (2008)	5EF		Stx2	Monoclonal scFv	Recombinant	*In vivo*
Strockbine et al. (1985); (Bitzan et al., 2009); Desoubeaux et al. (2013); Tzipori et al. (2004)	cαStx1 and cαStx2 (Shigamabs^®^)		Stx1 B; Stx2 A	Monoclonal IgG1	Chimeric	Clinical trials
Kimura et al. (2002); Yamagami et al. (2001); Moxley et al. (2017); López et al. (2010)	Urtoxazumb (TMA-15)		Stx2 b	Monoclonal IgG	Humanized	Clinical trials
Mukherjee et al. (2002a); Sheoran et al. (2003)	1G3; 2F10; 3E9; 4H9; 5C12; 5H8^c^; 6G3^c^		Stx2-a	Monoclonal IgG	Humanized	*In vivo*
Mukherjee et al. (2002b); Jeong et al. (2010)	2D9^b^; 5A4^a^; 10F4^a^; 15G2^a^; 15G9^b^		Stx1-b	Monoclonal IgM/IgG	Humanized	*In vivo*
Iwata et al. (2014)			Stx1 B	Monoclonal IgG/IgA	Recombinant	*In vitro*
Inoue et al. (2004)	5-5b rec		Stx1	Monoclonal Fab	Recombinant phage display	*In vitro*
Luz et al. (2015); Luz et al. (2018)	C11		Stx1; Stx2-b	Monoclonal scFv/Fab	Recombinant phage display	*In vivo*
Luz et al. (2021)	Fab F8:Stx2		Stx2	Monoclonal Fab	Recombinant phage display	*In vitro*
Tremblay et al. (2013)			Stx1; Stx2	Monoclonal VHH	Recombinant	*In vivo*
Mejías et al. (2016)			Stx2b	Monoclonal VHH	Recombinant	*In vivo*

a IgG.

b IgM.

c Stx2-b.

It is noteworthy that, once a biopharmaceutical is approved, it must be manufactured by the standards of purity and stability, as per regulatory agencies (Mohs and Greig, 2017), as well as being evaluated for its postmarketing safety, with the monitoring of possible adverse events (Tamimi and Ellis, 2009).

Herein, in this review, we intend to discuss antibodies against Stx and their trends and perspectives; for that, we employed as selection criteria the publications in PubMed between January 1, 1989 and October 1, 2021, with predilection given to renowned papers and review published in the last 10 years or so, with the tool “MeSH” using the terms “hemolytic uremic syndrome,” “thrombotic microangiopathy,” “Shiga toxin,” “Shiga-like toxins,” “verotoxin,” and “antibodies” in combination with the term “pathophysiology,” “causes,” “therapy,” and “treatment.”

We restricted our search to English publications. We first focused on review articles to provide more detail. Selected reports from the past 10 years but did not exclude important and highly cited older publications. For greater elucidation of these data, it searched the reference lists of selected articles identified by this search strategy and selected those relevant to the main topic. In the last decade, some broad and relevant reviews have been published; we highlighted (Keir et al., 2012; Page and Liles, 2013; Hall et al., 2017; Torres et al., 2018; Kakoullis et al., 2019; Joseph et al., 2020; Lingwood, 2020; Mühlen and Dersch, 2020; Hwang et al., 2021) those that covered different aspects of STEC such as epidemiology, diagnosis, serotypes, and HUS from pathogenicity to therapeutic options. Therefore, our decision was to focus on the therapeutic strategies using antibodies for Stx blockage. Below, we describe in detail all approaches used to develop anti-Stx antibodies.

### 2.2 Polyclonal Antibodies

The polyclonal antibody obtainment starts with the animal immunization, which in general is either mice or rabbits and consists of a purified antibody mixture from the immunized animal serum. The term polyclonal is the mixture of antibodies that comes from different lymphocyte clones and responds against different epitopes of the target toxin (Cosson and Hartley, 2016). pAbs have been used for neutralizing the toxicity of bacterial toxins since the golden age of microbiology (circa 1850), initially for diphtheria and tetanus, which was granted a Nobel prize to Emil von Behring in 1901 (Froude et al., 2011). Regarding Stx toxins, it was not different; indeed many groups started obtaining pAbs as soon as the first description of Stx intoxication was published (O'Brien et al., 1983). Several polyclonal strategies were attempted towards Stx, such as the use of immunized animal serum, bovine colostrum, and enriched IgY from avian eggs.

First, we highlight the work developed by O’Brien’s group, which has contributed to the knowledge of Shiga toxin since the 1980s. The Stx2-producing *E. coli* O157:H7 strain 86-24 was used to produce anti-Stx2 polyclonal rabbit antisera (Kokai-Kun et al., 2000). At rodent infection model, this rabbit anti-Stx2 pAb was administered in a single dose intraperitoneally. To determine the efficacy, fecal pellets were collected at different times postinfection and tested for toxin presence. From days 3–5 postchallenge, it was observed that the antiserum diminished the *E. coli* O157:H7 burden and extended the animal survival. However, by the seventh day, the level was similar to that of the control group (Mohawk et al., 2010).

Another important study was done by Takeda and his coworkers in characterizing Shiga toxin and investigating the prominent therapies to neutralize it. Initially, human serum anti-Stx1 and anti-Stx2 from HUS patients and those commercially available were tested against both toxins. Only one tested serum showed a neutralizing ability against all Stx1 (125 pg/ml); on the other hand, none of the tested serum neutralize Stx2 (Takeda et al., 1993; Adachi et al., 1998). Furthermore, the same group raised an antibody anti-Stx2 in rabbits, which showed *in vivo* protection against neurotoxicity caused by Stx2 when applied intrathecally within 2 h (Fujii et al., 1998).

In a different approach, immunoglobulin-rich bovine colostrum preparation containing a high titer of anti-Stx1 and anti-Stx 2 was tested in patients; however, the treated patient did not show a significant difference compared with the placebo-treated patient; also, HUS development or other possible infection complications were not analyzed by study subjects (Huppertz et al., 1999). On the other hand, some studies showed effectivity of colostrum containing anti-Shiga antibody administration to protect animals from death (Funatogawa et al., 2002; Seita et al., 2013). Similarly, colostrum IgG against Shiga toxin and bovine lactoferrin completely prevented lethality of *E. coli* O157:H7 in a weaned mouse model (Albanese et al., 2018). In addition, an early study in children showed that bovine colostrum is well tolerated, reduced the frequency of loose stools, and eliminated bacterial infection (Huppertz et al., 1999). In this line of thought, spray-dried and reconstituted hyperimmune bovine colostrum against Stx2 preserved the protective capacity against *E. coli* O157:H7 pathogenicity *in vitro* and *in vivo* models. In this regard, the hyperimmune bovine colostrum against Stx has been proposed as a preventive tool for STEC/EHEC infection control in bovine and humans (Garimano et al., 2021).

Egg yolk antibodies (IgY) can be obtained noninvasively by immunizing avians with specific antigens. IgY is a typical low molecular weight egg yolk antibody of birds, reptiles, amphibians, and lungfish (**Figure 2**) (Parma et al., 2011). It has interesting advantages such as obtaining better yields, higher antibody titers, and lower costs than IgG generation from the plasma of mammals (Svendsen et al., 1995; Sriram and Yogeeswaran, 1999). In this scenario, Parma and colleagues (Parma et al., 2011) used this know-how to produce anti-Stx2 IgY obtained from egg yolks of laying hens immunized with a recombinant Stx2B subunit. The anti-Stx2 IgY was able to recognize Stx2B and Stx2 under denatured conditions, as well as block the biological activity of Stx2 in Vero cells and protect mice from the Stx2 toxicity after antibody-toxin preincubation (Parma et al., 2011). Similarly, Fathi and his coworkers (Fathi et al., 2020) obtained IgY polyclonal antibodies from eggs after chicken immunization with EHEC O157:H7 supernatant (containing Stx1a and Stx2a). Mice challenge injected with 5LD_50_ of Stx showed that the concentration of 2 mg/mice IgY was able to reach 100% survival rate, while the entire control group died after 4 days. The raised antibody was able to neutralize Stx effects after preincubation, which suggests that it would be a promising prophylactic candidate. In 2013, an interesting paper from Feng and collaborators (Feng et al., 2013) discussed the use of IgY to neutralize Stx2e, known for causing porcine edema disease. *In vitro* and *in vivo* tests demonstrated neutralizing capacity upon Stx2e, but high antibody titers were needed to achieve that goal (Feng et al., 2013).

Even though such polyclonal antibodies showed promising neutralization abilities *in vitro* and *in vivo*, none of them got through further than the preclinical stage at the biopharmaceutical path, which we believe is because its animal origin, which could trigger anti-antibody effect, inactivates the therapeutic antibody before neutralizing the toxin activity (**Figure 3**). Moreover, the use of animal serum is a limiting process since it is limited by the size of the immunized animal. The discovery of lymphocyte immortalization to obtain hybridomas, which could grow in cell culture unlimitedly as well as secrete specific antibodies, was a breakpoint in antibody research.

### 2.3 Monoclonal Antibodies

Following the polyclonal antibody generation, Köhler and Milstein (Köhler and Milstein, 1975) discovered a method to immortalize mouse antibody-producing cells by fusing target-specific lymphocytes with myeloma cells. This was a landmark in the development of antibodies; with such remarkable consequences, they were awarded with the Nobel Prize for Physiology and Medicine in 1984. The antibodies obtained from this approach are specific to a unique epitope, giving high specificity to the developed molecule.

The first work to describe the generation of mAbs against Stx1 and Stx2 was performed by Padhye et al. (1989); however, only the Stx1 mAb prevented the death of mice exposed to Stx1 (Padhye et al., 1989). Following this, other groups also generate mAbs against Shiga toxins.

The mAb VTm1.1 was raised against Stx2 subunit B (epitope Ser30, Ser53, Glu56, Gln65, Asn68, and Asp69) from *Escherichia coli* O157:H7 (Nakao et al., 1999). The VTm1.1 mAb was able to neutralize the cytotoxic activity of Stx2 and subtypes derived from STEC isolates from patients but not those derived from animals (Nakao et al., 1999). The VTm1.1 molecule, later called TMA-15, after humanization and heterologous expression, was further analyzed as an anti-Stx2 promising blocker; it will be discussed below in the Recombinant Antibodies section of this review (Kimura et al., 2002). The same group also developed five different anti-Stx1 mAbs with affinity to subunit B, with the ability to neutralize Stx1 cytotoxicity *in vitro* (Nakao et al., 2002). Similarly, Ma et al. (2008) developed four novel mAbs anti-Stx2 (2 for Stx2A, and 2 for Stx2B), all of which have shown strong neutralization activities *in vitro* and *in vivo*. As the VTm1.1 antibody, the mAbs raised by Ma and colleagues were also transformed into scFv molecules and will be further discussed below.

Our group also developed and described mAbs against Stx toxins. The mAb anti-Stx1 (3E2) and anti-Stx2 (2E11) were generated using Stx1a and Stx2a toxoids as antigens. These mAbs showed neutralizing ability against either purified toxins or different Stx subtypes produced by STEC isolates (Rocha et al., 2012).

He and colleagues (He et al., 2013) tested the ability of different mAbs, namely, Stx2-1, Stx2-2, Stx2-4, Stx2-5, and Stx2-6 to neutralize Stx2 activity in Vero cells; only mAb Stx2-5 showed a significant neutralization activity in the cell-based assays. These mAbs were further characterized by Cheng and coworkers (Cheng et al., 2013), who tested the mAbs ability to protect mice from death. Challenge assays were performed by testing different doses of mAbs individually or combined. In contrast to the Vero cell toxin neutralization assays, mAbs Stx2-1 (anti-Stx2 Subunit A) and Stx2-2 (anti-Stx2) completely protected mice from death with only 5 µg/mouse of mAbs. MAb Stx2-5 (anti-Stx2 subunit B) provided the highest level of protection, showing full protection at 1 µg/mouse (Cheng et al., 2013; He et al., 2013).

Russo and colleagues (Russo et al., 2014) analyzed several parameters of mice infected with Stx2a and evaluated the neutralizing ability of the mAb 11E10. Besides protection from death, the mAb also prevented kidney damage, which is a promising feature, since HUS especially affects these organs (Russo et al., 2014).

Although promising, either polyclonal approaches or the hybridoma development results in molecules with an animal origin, and as therapeutic tool, the administration of these molecules could lead to a human antimurine antibody (HAMA) response, which may trigger several side effects besides the inactivation of the antibody effectivity.

In order to overcome this problem and make it possible for the discovered antibody to reach the human use stages, the researchers used the DNA recombinant technologies to improve the mAbs, whether by chimerization, humanization (by using transgenic animals which express the human IgG molecule, for example), or raising new rAbs at different formats, classes, or sources to obtain antibodies with lower immunogenicity.

### 2.4 Recombinant Antibodies

rAbs have numerous practical advantages over animal-derived molecules, such as control of antibody selection, format, production system, and storage. Indeed, in some approaches, rAbs can be obtained without animal immunization, so they can be selected in a less biased manner. Moreover, they can be designed to have a unique specificity or multiple ones, in different formats (**Figure 2**), besides being able to be produced unlimitedly and be stored safely (Cosson and Hartley, 2016). Thus, some groups worked to transform their pAbs and mAbs into these veritable molecules. Even though the rAbs format possibility is wider than presented here, we highlight only the strategies used against Shiga toxins.

Indeed, Ma et al. (2008) also tested their anti-Stx1 and anti-Stx2 mAbs and used them to obtain scFv molecules, which are smaller and easier to obtain from bacterial system. Their scFv were capable of neutralizing Stx toxicity, suggesting that there would be promising candidates against Shiga toxin-producing bacterial infection. Similarly, Luz et al. (2015) obtained scFv anti-Stx2 from a murine hybridoma previously characterized by Rocha et al. (2012). The anti-Stx2 scFv also showed neutralizing ability; however, because of its murine origin, in this work, the scFv was directed to function as a diagnostic tool. The problem of immunogenicity towards murine therapeutic antibodies led the researchers to search for other strategies.

#### 2.4.1 Chimeric Antibodies or Humanized Antibodies—IgG Format

The chimeric murine-human monoclonal antibodies (chi-Abs) were developed by Strockbine et al. (1985), comprising the variable regions of the murine Stx1- (B-subunit) or Stx2-neutralizing (A-subunit) antibodies 13C4 and 11E10 fused to the light chain of human IgG1; the chi-Abs were named cαStx1 and cαStx2 (Shigamabs^®^), which showed ability to neutralize Stxs in mice (Strockbine et al., 1985; Bitzan et al., 2009). In addition, they were well tolerated in healthy human volunteers when given as a single dose either separately or in combination (Dowling et al., 2005; Bitzan et al., 2009). Moreover, phase 2 clinical trial was performed enrolling STEC-infected children, showing the safety of its administration (Desoubeaux et al., 2013). Unfortunately, the efficacies of hybrid antibodies were often found to be lower compared with the murine parent antibodies (Tzipori et al., 2004). However, the major concern regarding chimeric mAbs is that they still retain murine IgG elements that could trigger HAMA effect.

To overcome this possibility, some groups rely on humanized antibodies, which combine the murine antibody complementary regions with a human framework and constant regions. The mAb VTm1.1 developed by Nakao et al. (1999) used this approach to produce TMA-15 (urtoxazumab) in cells Sp2/O-Ag14 (Yamagami et al., 2001; Kimura et al., 2002). The humanized TMA-15 was tested in postinfection experiments to prevent Stx2 binding to the B subunit, protecting mice from a lethal challenge with STEC when given within 24 h of infection, as well as able to reduce brain lesions and death in a gnotobiotic piglet model (Yamagami et al., 2001; Kimura et al., 2002; Moxley et al., 2017). Indeed, the postinfection administration approach is relevant since, in the clinic, the treatment is done after symptoms appear (Yamagami et al., 2001). This is one of a few anti-Stx antibodies to reach clinical trials phase I (**Figure 3**). The TMA-15 (urtoxazumab) was found to be well-tolerated and safe after being tested by intravenous application in a single randomized, intravenous, double-blind, placebo-controlled doses tested in healthy adults or pediatric patients with a confirmed STEC infection (López et al., 2010).

Another approach to obtain whole human IgG by hybridoma technology is by transgenic animal immunization (HumAb). To do that, the animals are genetically modified to produce human immunoglobulin (Ig) heavy and light chain loci, in general, the mouse is the animal used. Mukherjee and colleagues (Mukherjee et al., 2002a; Mukherjee et al., 2002b) developed a panel of hybridomas against both types of Shiga toxins. A total of 37 of specific anti Stx2 and 13 against Stx1 humanized antibodies (HumAbs) were isolated and tested. These HumAbs were able to prolong the survival of mice in an Stx1 toxicosis model (Mukherjee et al., 2002a) and higher survival of gnotobiotic piglets was observed when treated 48 h after challenge with an Stx2a-producing STEC strain (Sheoran et al., 2003). Interestingly, only 5C12 (anti-Stx2) protected piglets infected either with Stx1- or Stx2-producing strains (Jeong et al., 2010).

A different strategy was performed by Iwata et al. (2014), by developing an IgG/IgA hybrid against the B subunit of Stx1. The original molecules have a murine origin and were engineered to be expressed in Chinese hamster ovary cells (CHO-K1) as monomeric or dimeric formats. Both hybrid formats showed the ability to abolish the Stx cytotoxic effect on Vero cells; however, the dimeric hybrid was 10-fold more efficient than monomeric, suggesting that the binding site tetravalent characteristic may contribute to this neutralization efficacy.

#### 2.4.2 Antibody Selection by Phage Display—Fab and scFv Formats

In 2018, George P. Smith and Sir Gregory P. Winter, the researchers responsible for the development of the phage display technique were awarded with the Nobel Prize in Chemistry. George Smith first described the use of phage display in 1985 and the proof of concept for phage-displayed peptide libraries in 1990. Also in 1990, Sir Gregory Winter and his colleagues reported the expression of a functional and correctly folded antibody fragment in filamentous phages. Since then, this technology has been used for antibody research and development by organizations located around the world, resulting in more than 80 antibodies in clinical trials for different diseases (Kaplon and Reichert, 2019).

The pioneer group to obtain anti-Stx antibody fragments by this technology was Inoue and colleagues (Inoue et al., 2004). They generated anti-Stx1 Fab neutralizing antibodies fragments selected by phage display library prepared from anti-Stx1 hybridoma isolated genes (Nakao et al., 2002). These Fab were produced in a bacterial system, which differs from regular mAbs or whole IgG rAbs, consisting in a low-cost process (Luz et al., 2015).

Likewise, our group generated two anti-Stx2 Fab fragments by phage display using the human synthetic antibody library F and were expressed in bacterial systems (Persson et al., 2013; Luz et al., 2015; Luz et al., 2021). These Fab are fully human, which diminishes the possibility of antigenic reaction against them. The FabF8:Stx2 showed specificity only for Stx2 and protected human glomerular endothelial cells (HGEC) against Stx2 cytotoxicity (up to 83%), morphological alterations (90%), and apoptosis (93%). These protections were observed preincubating and to a lesser extent coincubating the toxin with FabF8:Stx2. In addition, this molecule was able to neutralize the cytotoxic effects of toxins secreted by Shiga toxin-producing *E. coli* strains harboring different *stx* gene subtypes (Luz et al., 2021). On the other hand, FabC11:Stx2 showed affinity to the B subunit (YTKYNDDTFT and GKIEFSKYNEDDTF epitopes) and cross-reacted with Stx1. It also protected both HGEC and human proximal tubular epithelial cells (HK-2) against the cytotoxicity and morphological alterations induced by Stx2. This protection, more prominent in HK-2 cells, has a dose-dependent behavior and occurred either pre- or coincubating the toxin with the antibody (Luz et al., 2018). Moreover, FabC11:Stx2 protected mice from death and kidney damage when administered after preincubation (Luz et al., 2018).

Another rAbs format were also described against Stx, such as the scFv anti-Stx1 and anti-Stx2 developed by Neri et al. (2011). The antibody fragments were selected from a human naive library AIM-5 (Morino et al., 2001) against both toxins, but just the scFv anti-Stx1 was able to neutralize the toxin; however, it was the first monomeric antibody described showing a neutralizing ability towards Stx 1 toxicity.

#### 2.4.3 Nanobodies—VHH Format

The VHH format is one of the possible single-domain antibodies (nanobodies), which are small antigen-binding fragments generated from heavy chains only present in camelid antibodies, which do not express light chains (**Figure 2**). Unlike mouse heavy variable chain, the VHH are in general soluble and stable for *in vitro* production (Holliger and Hudson, 2005). To overcome or at least reduce the immunogenicity problem related to animal origin, these fragments are engineered with the mutation that minimizes the immunogenic and hydrophobic residues followed by a presentation in display technique, such as the phage display previously discussed here.

One group used this approach to obtain anti-Stx nanobodies. Tremblay and his coworkers (Tremblay et al., 2013) raised anti-Stx1 and anti-Stx2 VHH and created VHH-based heterodimers as a toxin-neutralizing agent. This single toxin-neutralizing agent consists in a double-tagged VHH heterotrimer (one Stx1-specific VHH, one Stx2-specific VHH, and one Stx1/Stx2 cross-specific VHH). Their engineered antibody-based strategy was effective in preventing all Stx1- and Stx2-related symptoms when coadministered with an effector antibody and opened new therapeutic approaches to managing the disease (Tremblay et al., 2013).

Likewise, a trivalent VHH molecule (two copies of anti-Stx2B VHH and one anti-seroalbumin VHH) was raised by Mejías et al. (2016), which has a higher half-life and showed high therapeutic activity against Stx2 toxicity in three different mouse models (single i.v. Stx2 lethal dose, several i.v. incremental Stx2 doses, and intragastric STEC infection), suggesting being a promising option to treating STEC infections to prevent or ameliorate HUS outcome (Mejías et al., 2016).

## 3 Trends

Antibodies are versatile molecules, and their pharmaceutical generation can range from polyclonal plasma from infected animals, monoclonal antibody-secreting hybridoma, to recombinant antibody fragments. Indeed, innovative recombinant DNA technologies have enhanced the murine mAb clinical efficacy and led to regulatory approvals for immunoglobulin and monovalent antibody fragment molecules. Certainly, these molecules have been the focus of the strengths of the global biopharmaceutical industry in order to convey innovative antibody therapeutics to patients for several diseases, mainly cancer, some immunological disorders, and recently for SARS-Cov-2. However, this scenario is not the same for toxins produced by bacteria in general, in which we may include antibodies against Stx.

The worst outcomes of Stx infections are HC and HUS, and for that, the use of antibiotics is a debating issue, while some antibiotics such as beta-lactams and trimethoprim/sulfamethoxazole may be detrimental, others appear to be safe and can prevent the development of HUS. Importantly, fosfomycin appears to be the antibiotic with the most positive results from clinical studies and may be able to prevent HUS development, especially if administered within the first 2 or 3 days from diarrhea onset. Likewise, fluoroquinolones have also shown positive outcomes in clinical studies, despite demonstrating unfavorable results in *in vitro* studies. Other agents, such as colistin, gentamycin, and rifamycin, have shown promising results in *in vitro* studies and require further evaluation (Kakoullis et al., 2019). An ideal STEC infection antibiotic therapy should kill or inhibit the bacteria without inducing Stx expression at any concentration. In this sense, in combination with neutralizing antibodies to Stx1 and Stx2, the tigecycline-antibody treatment fully protected Vero cells from Stx toxicity, even when the STEC bacteria and the Vero cells were cultured together (Skinner et al., 2015). However, there is still a need for preventive early therapy of STEC infections to avoid HUS development. In this regard, antibodies are an excellent and versatile approach.

Most of the antibodies raised either polyclonal, or monoclonal, or recombinant presented in this review rely on bench studies, mainly *in vitro*, and some were tested in mice and piglets showing that they mainly differ in their protective efficacy and/or their specificity to Stx subtypes, but they are promising tools. It’s worth mentioning two mAbs: urtoxazumab (TMA-15) and Shigamabs^®^ (cαStx1 and cαStx2) which were tested in humans. TMA-15 was found to be well-tolerated and safe in healthy adults or pediatric patients in intravenous applications (López et al., 2010). The dose-related safety was not noted and anti-urtoxazumab antibodies were not detected, suggesting low immunogenicity, but further investigation is needed of urtoxazumab to assure security. Shigamabs^®^ was evaluated in forty-five children with macroscopic bloody diarrhea for less than 36 h, and STEC-positive stools were randomized into three groups receiving either 1 or 3 mg/kg of each antibody or placebo. In general, the adverse events were mild and transient and equally distributed between groups. Three serious adverse events, including two HUS cases, were reported, and all were considered unrelated to the drug study. Moreover, one patient developed an asymptomatic immune response against cαStx2. Shigamabs^®^ thus appeared safe and well-tolerated in STEC-infected children (Desoubeaux et al., 2013). The small sample size made it difficult to infer any trends in efficacy in this study. However, different clinical trial phases must still confirm their efficacy. As described for vaccine trials, therapy employing antibodies will face the same problems: while phase II clinical trials can be carried out, the availability of patients with STEC infections for phase III trials is limited, besides, the time of presentation at the physician or in hospital will most likely be after the onset of bloody diarrhea or late stages of watery diarrhea, making an early intervention difficult (Mühlen and Dersch, 2020).

## 4 Perspectives

Based upon the *in vivo* experiments so far presented, passive antibody transfer is a viable therapeutic option for STEC infection, since Stx seems to be delivered at a continual low dose. However, the knowledge about the time when the Stx enters the bloodstream and the Stx levels in the blood and infected tissues is scarce. Therefore, a very critical point for that with Stx antibodies that has to be considered for successful therapy is the time point and dosage of antibody administration: infected patients might be protected against the development of HUS when the antibodies are given shortly after the onset of diarrhea. Also, for these small molecules such Fab, there is a necessity to increase its half-life by conjugating them to carriers.

Another point to be considered is even though there are some animal models which mimic some STEC infection characteristics, in general, mice and piglets do not develop either bloody diarrhea or HUS as humans, making it hard to prove the results indicating that protective effect of Stx-specific antibodies cannot easily be transferred to humans. Furthermore, as Shiga toxin-producing bacterial infections occur as outbreaks, it is difficult to enroll volunteers for clinical trials.

In summary, there are still many challenges to overcome in order to reach the desirable anti-Stx neutralizing molecules; however, one thing is indisputable, antibodies are the closest known molecules to a perfect weapon against these powerful toxins.

## Author Contributions

Conceptualization: IMH, RMFP, and DL. Data curation: IMH, RLF, and CH. Writing—original draft preparation: IMH, FS, CH, RLF, MMA, RMFP, and DL. Writing—review and editing: RMFP and DL. Supervision: DL and RMFP. All authors have read and agreed to the published version of the manuscript. All authors listed have made a substantial, direct, and intellectual contribution to the work and approved it for publication.

## Funding

This work was supported by São Paulo Research Foundation (FAPESP-2015/17178-2 and FAPESP-2018/13895-0 to RP and 2013/03160-9 and 2019/24276-1 to DL) and by the “National Agency for Promotion of Science and Technology” (grant number ANPCYT-PICT 2017-0617 to MA), the “University of Buenos Aires” (grant number UBACYT-20020170200154BA to MA), and the “National Scientific and Technical Research Council” (grant number CONICET: PUE 0041 to MA). IH, a master student is a CAPES-recipient fellow (CAPES-PROEX 88.887.509.845/2020-00; RLF, an undergraduate student was a recipient of a fellowship from FAPESP (2018/24659-5) and Fundação Butantan, and currently, she is a recipient of a fellowship from the Brazilian National Council (PIBIC-CNPq). CH, a PhD student is a FAPESP-recipient fellow (2017/17213-8). RP received a fellowship from the National Council of Scientific and Technological Development (CNPq 303969/2017-2).

## Conflict of Interest

The authors declare that the research was conducted in the absence of any commercial or financial relationships that could be construed as a potential conflict of interest.

The handling editor declared a shared affiliation with one of the authors/with several of the authors, FS and MA, at the time of review.

## Publisher’s Note

All claims expressed in this article are solely those of the authors and do not necessarily represent those of their affiliated organizations, or those of the publisher, the editors and the reviewers. Any product that may be evaluated in this article, or claim that may be made by its manufacturer, is not guaranteed or endorsed by the publisher.
